# Intratumoral calcifications in pancreatic neoplasms on unenhanced CT: frequency and diagnostic implications

**DOI:** 10.1007/s11547-025-02142-4

**Published:** 2025-11-03

**Authors:** Riccardo De Robertis, Maria Chiara Brunese, Nicolò Cardobi, Flavio Spoto, Francesca Pasquazzo, Beatrice Mascarin, Fabrizio Urraro, Alfonso Reginelli, Luca Brunese, Salvatore Cappabianca, Mirko D’Onofrio

**Affiliations:** 1 Department of Diagnostic and Public Health, University of Verona, Verona, Italy; 2 Department of Radiology, Azienda Ospedaliera, Universitaria Integrata Verona, Piazzale L.A. Scuro 10, 37134 Verona, Italy; 3 Department of Medicine and Health Science, University of Molise, 86100 Campobasso, Italy; 4 University of Campania “L. Vanvitelli”, Naples, Italy; 5 Department of Life Sciences, Health and Health Professions, “Link Campus University”, Rome, Italy; 6 Department of Precision Medicine, University of Campania Luigi Vanvitelli, Naples, Italy

**Keywords:** Pancreas, computed tomography, magnetic resonance imaging, pancreatic cyst, pancreatic cystic neoplasm

## Abstract

**Purpose:**

Serous cystadenomas (SCAs), solid pseudopapillary neoplasms (SPNs), neuroendocrine neoplasms (NENs), and mucinous cystic neoplasms (MCNs) are pancreatic tumors that frequently develop calcifications. Identifying the presence and pattern of calcifications on unenhanced CT scans can significantly aid radiologists in differential diagnosis.

**Methods:**

Patients were included if they had a confirmed diagnosis through pathology or endoscopic ultrasound and MRI follow-up for at least one year. Exclusion criteria were the absence of CT imaging and multiple pancreatic lesions. Two radiologists independently reviewed unenhanced CT scans to assess lesion location, size, presence of calcifications, and calcification patterns, categorized as Type 1 (punctate), Type 2 (curvilinear/elongated), and Type 3 (coarse). Statistical analysis was performed using Fisher’s test for categorical variables, Kruskal–Wallis and Mann–Whitney tests for numerical variables, and logistic regression models to assess the impact of calcification patterns on diagnosis. Sensitivity, specificity, accuracy, and AUC-ROC were calculated for predictive models.

**Results:**

311 patients (mean age 61 ± 14 years; 56.9% female) were included. Calcifications were present in 27.7% of cases. Calcified NENs and SPNs were more frequently in the body/tail (*p* = 0.003), and calcified NENs were larger than non-calcified ones (*p* < 0.001). Punctate calcifications were most common in NENs, while coarse calcifications predominated in SCAs, decreasing the likelihood of a NEN diagnosis and increasing the probability of SCA. The AUC-ROC values were 0.891 for NENs and 0.986 for SCAs.

**Conclusions:**

Approximately 30% of pancreatic tumors exhibit calcifications. Punctate intratumoral calcifications are more indicative of NENs, whereas coarse calcifications strongly suggest SCAs, influencing the differential diagnosis.

## Highlights

- Calcifications can be found in around 30% of pancreatic neuroendocrine neoplasms (NENs), serous cystadenomas (SCAs), mucinous cystic neoplasms (MCNs), and solid pseudopapillary neoplasms (SPNs).
- Calcifications depicted at unenhanced CT may help differentiate pancreatic neoplasms.
- Intratumoral punctate calcifications are more frequent in NENs
- Coarse intratumoral calcifications are more common in SCAs and decrease the likelihood of NEN.

## Introduction

Pancreatic neoplasms have a variable biological aggressiveness, including aggressive forms, such as ductal adenocarcinoma (PDAC) and neuroendocrine carcinoma, malignant lesions with a better prognosis, such as solid pseudopapillary neoplasms (SPNs) and neuroendocrine tumors (NENs), premalignant or potentially malignant lesions such as intraductal papillary mucinous neoplasms (IPMNs) and mucinous cystic neoplasms (MCNs), and almost invariably benign lesions like serous cystadenoma (SCA). As such, imaging characterization is the cornerstone of patient management. As computed tomography (CT) has come to play a central role in medical care, especially in the emergency setting, there has been an increase in the frequency of incidental diagnoses of pancreatic neoplasms at CT performed for unrelated reasons [1]. Unenhanced CT is usually unable to characterize pancreatic neoplasms, as it is mainly related to their enhancement pattern; moreover, distinguishing between a solid and a cystic pancreatic neoplasm may be challenging at unenhanced CT, and magnetic resonance (MR) examination is commonly necessary for a final diagnosis [2].

Pancreatic calcifications, easily depicted at unenhanced CT, may arise from many etiologies and are commonly associated with chronic pancreatitis. Less frequently, calcifications can be found within pancreatic neoplasms. According to a previous study [3], SCAs, SPNs, neuroendocrine neoplasms (NENs), and MCNs are the pancreatic tumors that more commonly contain calcifications, with a reported incidence rate ranging from 15 to 30%; on the other hand, PDACs and IPMNs do not generally calcify [4].

It is unclear whether the presence and the pattern of calcifications could help characterize pancreatic neoplasms; this could be helpful for incidentally detected pancreatic neoplasms at CT examinations performed for unrelated reasons, as it may optimize patients’ management, e.g., directly addressing suspected cystic pancreatic neoplasms to MR examination.

However, the impact of clarifying the patterns of calcifications of pancreatic lesions at standard CT scan has not been clearly investigated. Contrast-enhanced MRI and CT scans are the gold standard for defining pancreatic neoplasms [5, 6]. Still, we have experienced challenging cases where the classification of calcification patterns has helped complete the diagnosis. Furthermore, baseline CT scan can be commonly found in the patient's medical history, and its re-evaluation of any incidental findings may improve the diagnostic power of radiologists according to our radiological criteria for tumor characterization.

This study aimed to evaluate the incidence of calcifications and the pattern among pancreatic NENs, SCAs, MCNs, and SPNs at unenhanced CT and how they can aid in the differential diagnosis.

## Materials and methods

Our Institutional Review Board approved this retrospective study and waived the need for specific informed consent.

The study is compliant with Regulation (European Union) 2016/679 of the European Parliament, of the Council of April 27, 2016, and according to Italian law (resolution March 1, 2012, Gazzetta Ufficiale No. 72 of March 26, 2012) on the use and protection of personal data. Ethics approval and informed consent were not required, owing to the retrospective design, the use of anonymized data, and the noninterventional nature of the study.

### Study population

We performed a retrospective research on our whole database containing all the information concerning CT, MRI, and US performed at our Institution from January 2017 to January 2024. The database was completed with clinical history and pathological reports. We identified all consecutive cases reporting NENs, SCAs, MCNs, and SPNs, as well as any eventual synonyms. “Adenocarcinoma” and “IPMNs” have not been included in the research.

Inclusion criteria were a) final diagnosis established by pathology (fine-needle aspiration or surgical resection) or typical findings at endoscopic ultrasound (EUS) and MR follow-up ≥ 1 year.

**Fig. 1:**
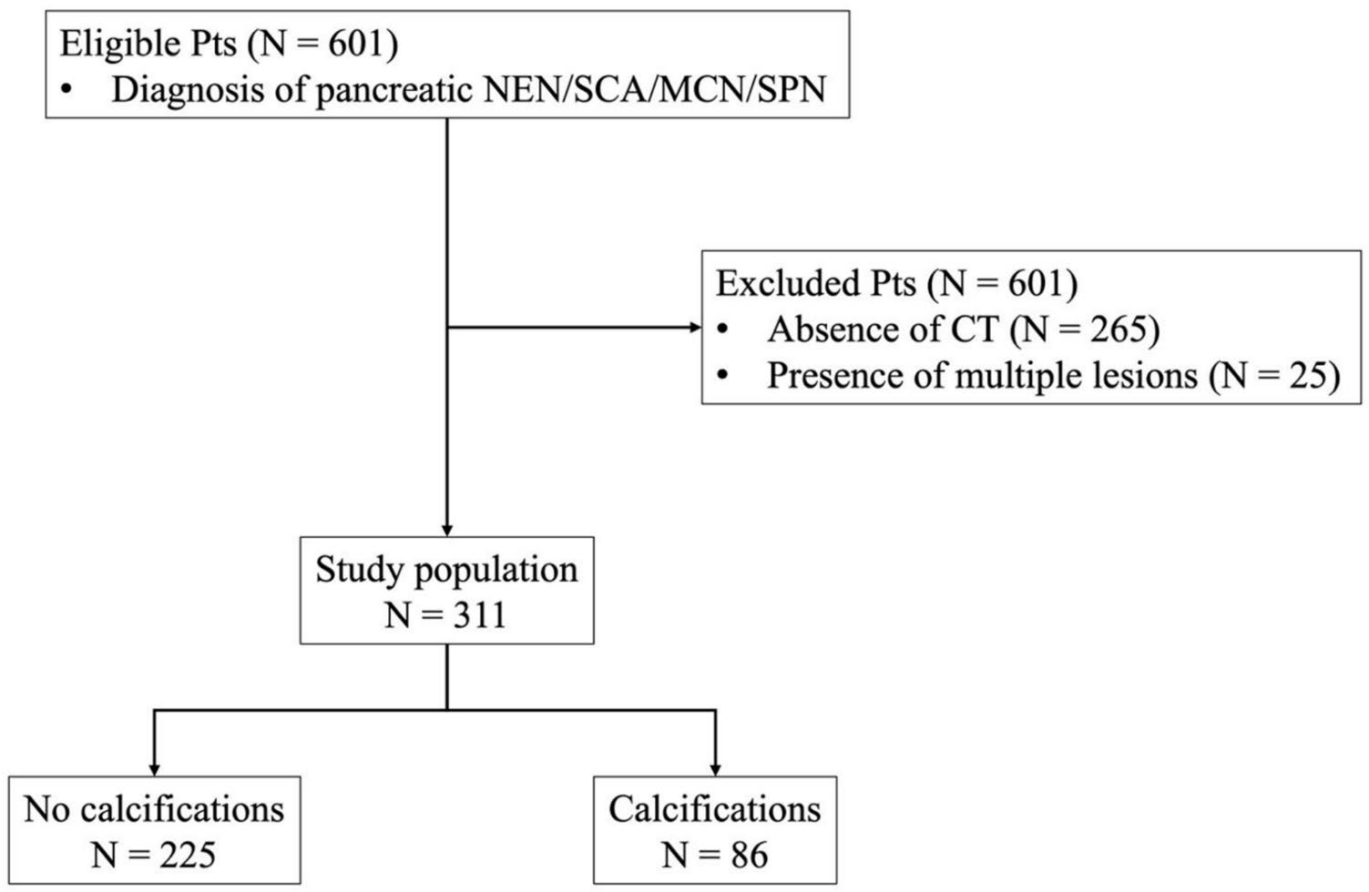
Patients’ selection

Exclusion criteria were a) absence of CT examination; b) presence of multiple lesions (Fig. 1). Demographic features and pathological and follow-up findings were retrieved from patients’ medical reports.

### Image analysis

CT examinations were performed on a 64-row scanner (Brilliance 64, Philips Healthcare). Patients were asked to fast at least 6 h before the examination and received 500 mL of water 10–20 min before imaging. A quadriphasic (unenhanced, late arterial, portal venous, and delayed phases) examination was performed. Unenhanced, late arterial, and delayed scans comprised the upper abdomen, whereas the portal venous scan examined the whole abdomen. For contrast-enhanced imaging, all patients received a weight-based amount of iodinated contrast agent through an antecubital vein of the arm, using an automatic power injector at a flow rate of 3–4 mL/s, followed by a 50-mL saline flush. The scan timing was determined using bolus tracking. Late arterial and portal venous phase scanning started 15 and 60 s after the attenuation threshold of 150 HU was reached; the delayed phase was acquired ≥ 180 s after the start of contrast medium administration [7].

All the images, including baseline CT, were acquired with a 64 mm × 0.625–1.25 mm collimation. The reconstruction thickness was 1–2 mm, with a 0.5- to 1-mm interval.

**Fig. 2:**
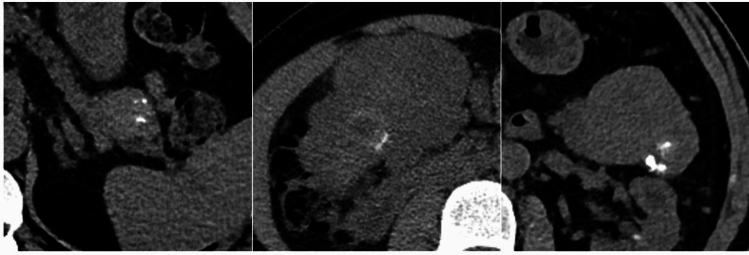
Patterns of calcifications. **a** type 1, intratumoral punctate calcifications; **b** type 2 curvilinear/elongated calcifictaions; c type 3, coarse intratumoral calcifications

Two radiologists (R.D.R. and M.D.O., with 10 and 25 years of experience in abdominal radiology) reviewed by consensus the unenhanced phase of each CT examinations included in the study to evaluate the following features: a) lesion location (pancreatic head vs. body/tail); b) lesion size; c) presence of calcifications; d) pattern of calcifications (type 1, i.e., punctate calcifications; type 2, i.e. curvilinear/elongated calcifications; type 3, i.e. coarse calcifications; Fig. 2).

### Statistical analysis

Comparison between groups (i.e., NENs vs. SCAs vs. MCNs vs. SPNs, and tumors without and with calcifications) was performed using Fisher’s exact test for categorical variables, and Kruskal–Wallis or Mann–Whitney U tests for numerical variables. Multiple logistic regression analyses were conducted to ascertain the effect of patients’ age and sex, tumor locations and size, and calcification pattern on the diagnosis of each tumor histotype. Sensitivity, specificity, accuracy, and area under the receiver operator curve (AUC-ROC) of the models were calculated. *P* values ≤ 0.05 were considered to be statistically significant. Statistical analysis was performed using JASP, version 0.19.1 [3].

## Results

### Study population

601 patients were eligible; 265 were excluded due to the absence of CT examinations; 25 were excluded for multiple lesions.

The final study cohort comprised 311 patients: 177 females (56.9%) and 134 males (43.1%), with a mean age of 61 years (standard deviation—SD, 14 years). NENs were the most frequent lesion (52.7%), followed by SCAs (26.4%), MCNs (13.8%), and SPNs (7.1%). Most lesions were found in the pancreatic body/tail (56.3%). The mean lesion size was 35.3 ± 24.1 mm. Calcifications were found in 27.7% of the lesions and were more frequent among SPNs (68.2% of cases), followed by NENs (33.5%), SCAs (15.9%), and MCNs (7%). Details on the study cohort and results of Fisher’s and Kruskal–Wallis tests are presented in Table 1.

**Table 1:** Characteristics of the study population and results of Fisher’s and Kruskal–Wallis tests. Categorical variables are presented as the number of cases (percentage); numerical variables are presented as the mean value ± standard deviation

Variable	Total N = 311	NEN N = 164	SCA N = 82	MCN N = 43	SPN N = 22	*P* value
Sex Male Female	134 (43.1%) 177 (56.9%)	99 (60.4%) 65 (39.6%)	28 (34.1%) 54 (65.9%)	3 (7%) 50 (93%)	4 (18.2%) 18 (81.8%)	< 0.001
Age (ys)	61 ± 14	61 ± 12	66 ± 11	56 ± 13	42 ± 16	< 0.001
Location Head Body/tail	136 (43.7%) 175 (56.3%)	72 (43.9%) 92 (56.1%)	52 (63.4%) 30 (36.6%)	3 (7%) 50 (93%)	9 (40.9%) 13 (59.1%)	< 0.001
Size (mm)	35.3 ± 24.1	32.2 ± 22.8	33.8 ± 18.5	41.8 ± 24.1	51 ± 39.9	0.001
Calcifications No Yes	225 (72.3%) 86 (27.7%)	109 (66.5%) 55 (33.5%)	69 (84.1%) 13 (15.9%)	40 (93%) 3 (7%)	7 (31.8%) 15 (68.2%)	< 0.001

ys, years; mm, millimeters; NEN, neuroendocrine neoplasm; SCA, serous cystadenoma; MCN, mucinous cystic neoplasm; SPN, solid pseudopapillary neoplasm

### Analysis of calcifications

No significant differences were found between calcified and noncalcified tumors for patients’ sex and age (*p* = 0.451 and 0.355, respectively). There was a significant difference between groups for location: tumors with calcifications were more frequently found in the pancreatic body/tail than in the pancreatic head (69.8% vs. 30.2%, *p* = 0.003); subgroup analysis (Table 2) revealed that calcified vs. noncalcified NENs and SPNs had significant differences in terms of location (p = 0.017 and 0.004, respectively), with a higher rate of calcified lesions in pancreatic body/tail tumors. Calcified tumors were larger than non-calcified ones (43.6 ± 30.3 vs. 32.1 ± 20.4, p < 0.001); subgroup analysis (Table 2) revealed a significant size difference only for NENs (*p* < 0.001).

**Table 2:** Subgroup analysis and results of Fisher’s and Mann–Whitney exact test. Categorical variables are presented as the number of cases (percentage of the total cases); numerical variables are presented as the mean value ± standard deviation

Histotype	Variable	Calcifications		*P* value
		No	Yes	
NEN	Location Head Body/tail	55 (76.4%) 54 (58.7%)	17 (23.6%) 38 (41.3%)	0.017
	Size (mm)	27.8 ± 19.2	40.9 ± 26.7	< 0.001
SCA	Location Head Body/tail	46 (88.5%) 23 (76.7%)	6 (11.5%) 7 (23.3%)	0.159
	Size (mm)	32.5 ± 17.8	40.6 ± 21.3	0.144
MCN	Location Head Body/tail	3 (100%) 37 (92.5%)	0 (0%) 3 (7.5%)	0.623
	Size (mm)	40.5 ± 24.3	59.7 ± 14.6	0.059
SPN	Location Head Body/tail	6 (66.7%) 1 (7.7%)	3 (33.3%) 12 (92.3%)	0.004
	Size (mm)	46.3 ± 18.7	53.3 ± 47.2	0.832

NEN, neuroendocrine neoplasm; SCA, serous cystadenoma; MCN, mucinous cystic neoplasm; SPN, solid pseudopapillary neoplasm; mm, millimeters

**Fig. 3:**
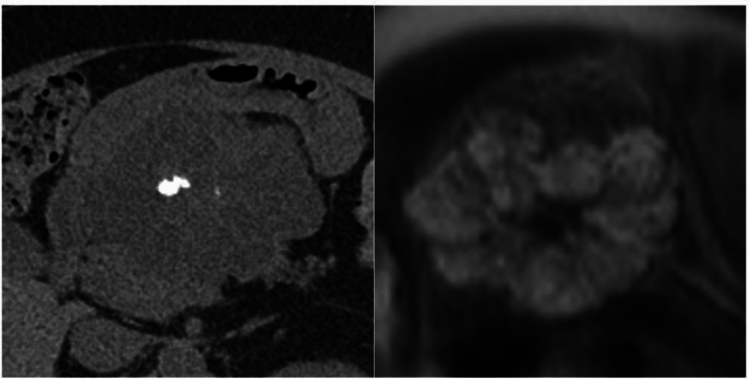
57-year-old woman with serous cystadenoma. **a** CT image showing a coarse internal calcifictaions (type 3 pattern); **b** on MR, the lesion presents the typical “honeycomb” appearance, with T2-hyperintense content

**Fig. 4:**
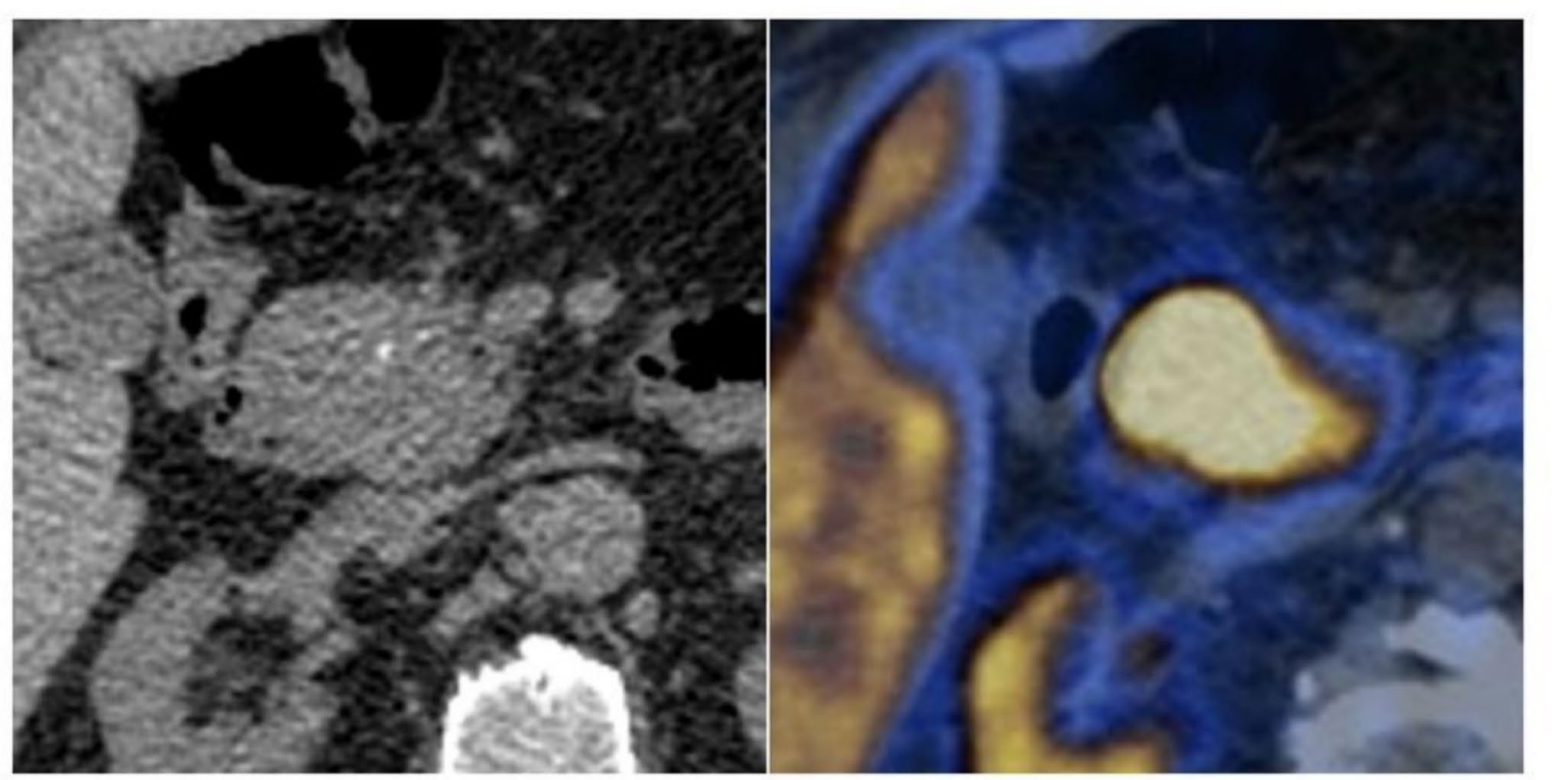
49-year-old man with pancreactic neuroendocrine neoplasm. **a** CT image showing intratumoral punctate calcifications (type 1 pattern); **b** at PET-CT scan the lesion intense ^68^Gallium uptake

Type 1 calcifications were more commonly found (38.4%), followed by type 3 (37.2%) and type 2 (24.4%). There was a significant difference between tumor histotypes (*p* = 0.002), with the highest incidence of type 3 calcifications among SCAs (Fig. 3) and a higher incidence of type 1 calcifications within NENs (Fig. 4). Data are reported in Table 3.

**Table 3:** Patterns of calcifications and results of Fisher’s exact test

Variable	Total N = 86	NEN N = 55	SCA N = 13	MCN N = 3	SPN N = 15	*P* value
Calcification pattern 1 2 3	33 (38.4%) 21 (24.4%) 32 (37.2%)	26 (47.3%) 15 (27.3%) 14 (25.4%)	1 (7.7%) 0 (0%) 12 (92.3%)	1 (33.3%) 1 (33.3%) 1 (33.3%)	5 (33.3%) 5 (33.3%) 5 (33.3%)	0.002

NEN, neuroendocrine neoplasm; SCA, serous cystadenoma; MCN, mucinous cystic neoplasm; SPN, solid pseudopapillary neoplasm

1: punctate calcifications; 2:curvilinear/elongated calcifications; 3:coarse calcifications

### Logistic regression analysis

**Fig. 5:**
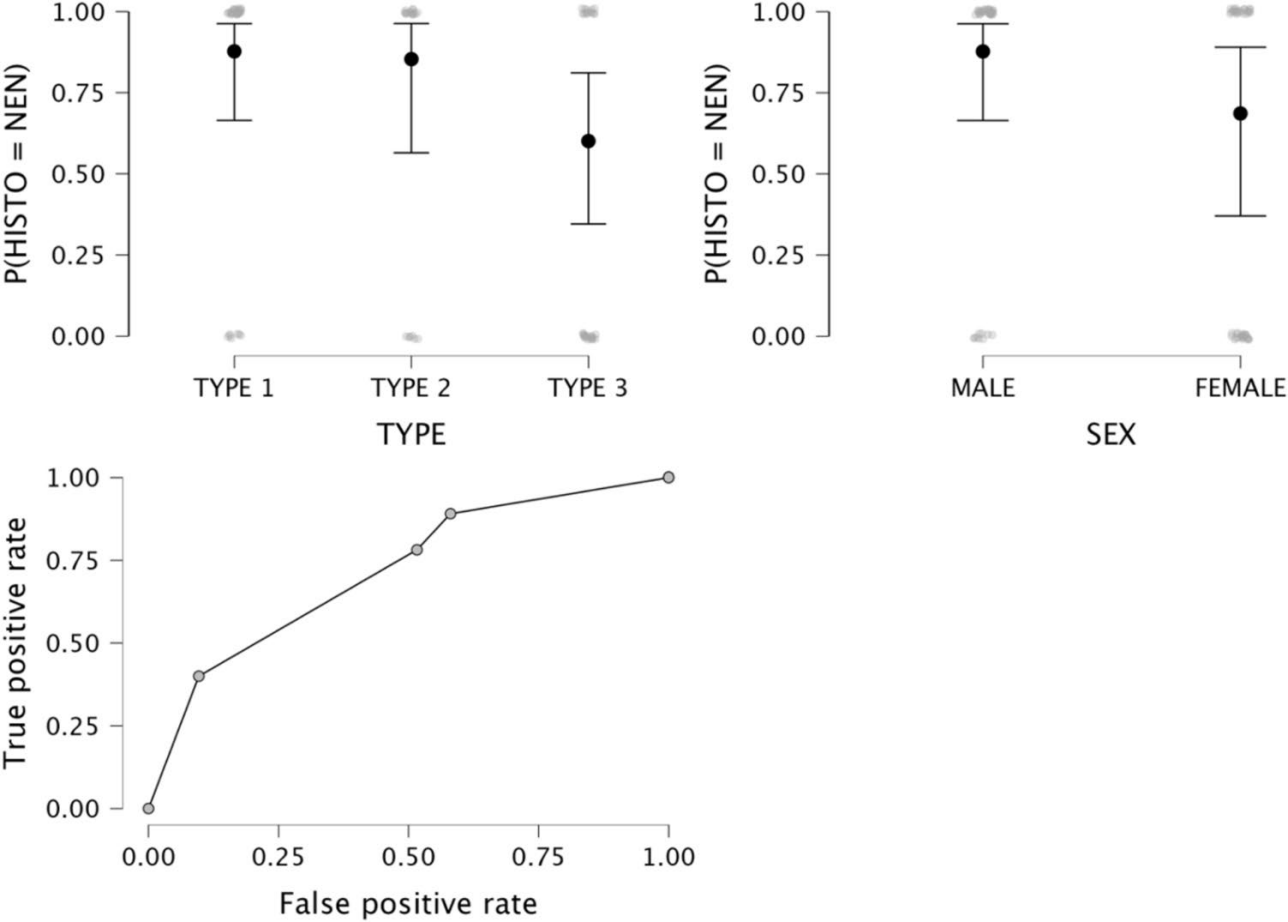
Logistic regression model for the differentiation of NENs from other histotypes. **a** and **b** estimate plots for calcifications patterns and sex; **c** ROC curve

**Fig. 6:**
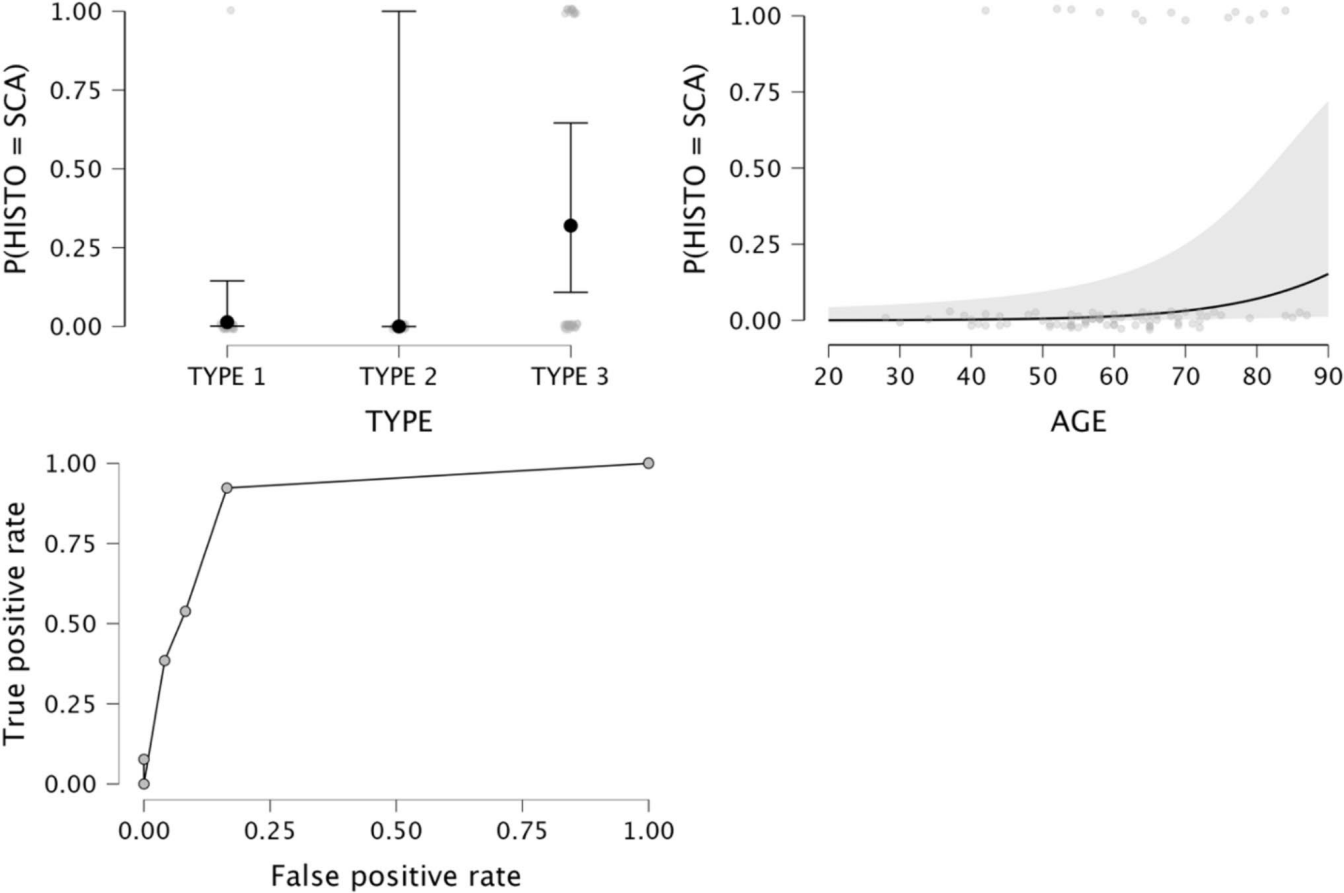
Logistic regression model for the differentiation of SCAs from other histotypes. **a** and **b** estimate plots for calcifications patterns and age; **c** ROC curve

Data on the logistic regression models are provided in Table 4. The logistic regression model for MCNs was not statistically significant (*p* = 0.216). The logistic regression model for NENs was statistically significant, χ2 (79) = 16, *p* = 0.014. The model correctly classified 89.1% of cases. Type 3 calcifications and female sex were significant predictor variables (*p* = 0.008 and 0.026, respectively), with odd ratios of 0.21 and 3.1; sensitivity, specificity, accuracy, and AUC-ROC of the model were 89.1%, 41.9%, 72.1%, and 0.891, respectively (Fig. 5). Statistical significance was also found for the logistic regression model for SCA, χ2 (79) = 31.1, *p* < 0.001. The model correctly classified 46.2% of SCAs. Patient’s age and type 3 calcifications were significant predictor variables (*p* = 0.027 and 0.004, respectively), with odds ratios of 1.1 and 33.8, respectively; sensitivity, specificity, accuracy, and AUC of the model were 46.2%, 94.5%, 87.2%, and 0.945, respectively (Fig. 6). The logistic regression model for SPNs was statistically significant, χ2 (79) = 17.2, *p* = 0.009. The model correctly classified 33.3% of cases. Patients’ age was the only significant predictor variable (p = 0.002), with an odds ratio of 0.9; sensitivity, specificity, accuracy, and AUC of the model were 33.3%, 98.6%, 87.2%, and 0.986, respectively.

**Table 4:** Logistic regression models

Model	*P* value	Sensitivity	Specificity	Accuracy	AUC-ROC
NEN vs. other SCA vs. other MCN vs. other SPN vs. other	0.014 < 0.001 0.216 0.009	89.1% 46.2% n.a 33.3%	41.9% 94.5% n.a 98.6%	72.1% 87.2% n.a 87.2%	0.891 0.945 n.a 0.986

NEN, neuroendocrine neoplasm; SCA, serous cystadenoma; MCN, mucinous cystic neoplasm; SPN, solid pseudopapillary neoplasm; AUC-ROC, area under the receiver operator curve; n.a., not assessed

## Discussion

The first aim of this study was to evaluate the incidence of calcifications and the pattern of calcifications among pancreatic NENs, SCAs, MCNs, and SPNs at unenhanced CT. Calcifications were found in 27.7% of the lesions and were more frequent among SPNs (68.2% of cases), followed by NENs (33.5%), SCAs (15.9%), and MCNs (7%). Overall, tumors containing calcifications were larger than non-calcified ones (43.6 ± 30.3 vs. 32.1 ± 20.4, *p* < 0.001), even though this difference was significant only for NENs (*p* < 0.001). Type 1 calcifications were more commonly found (38.4%), followed by type 3 (37.2%) and type 2 (24.4%). Secondly, we aimed to evaluate whether the pattern of calcifications could aid in the differential diagnosis between tumor histotypes. Type 1 calcifications were more frequent among NENs, while type 3 calcifications were more frequent among SCAs. Logistic regression analysis demonstrated that the presence of type 3 calcifications was associated with a decreased likelihood of NEN diagnosis and an increased likelihood of SCA diagnosis.

The incidence of calcified pancreatic neoplasms is unknown due to differences in imaging methods. The most common solid pancreatic tumor, i.e., PDAC, characteristically does not calcify [8, 9]; although not commonly seen, dystrophic calcifications may develop within mucin plugs contained in the dilated branches of IPMNs [10]; as such, these calcifications do not constitute part of the neoplastic mass. The imaging appearance of pancreatic NENs is broad, depending on size and grade of differentiation; calcifications have been reported in 15–20% of pancreatic NENs [3, 11, 12], and they occur more frequently in non-hyperfunctioning NENs, which are more likely to outgrow their blood supply, resulting in necrosis and dystrophic calcifications [13]. SCA is composed of thin-walled cysts containing serum. The most common appearance is a micro/macro cystic lesion, with thin septa oriented centrally toward a fibrovascular scar, which includes a coarse calcification in up to 30% of cases [3, 4, 14, 15]. Calcifications are common in SPNs and are thought to be dystrophic following intratumoral hemorrhage, typical of this tumor [16]. Large SPNs may contain calcifications in up to 65% of cases [3, 12, 14, 17, 18]. MCNs present calcifications in 15–25% of cases [3, 12], and the mechanism of calcification is uncertain and probably dystrophic; calcifications have been regarded as a predictive feature of higher malignancy, but further analysis is needed to confirm this data [19, 20].

The incidental detection of a pancreatic lesion at CT examinations performed for unrelated reasons may pose a relevant diagnostic dilemma. While uncommonly found, with a reported incidence of around 4% [1], pancreatic incidentalomas harbor a significantly higher malignancy rate than those detected in other organs. Proper and timely characterization is necessary through MRI integrated with CT features [21]. While the presumptive diagnosis of noncalcified tumors on unenhanced CT mainly relies on nonspecific findings, such as tumor attenuation, margins, and presence of ductal dilatation, this study demonstrated that the pattern of calcifications, which are on the opposite easily detected at unenhanced CT, may be helpful to address the differential diagnosis, in particular, to distinguish between NENs, SCAs, and other tumor histotypes. Current evidence indicates that serous cystadenoma (SCA) is correctly diagnosed in only 33% of cases, underscoring that the diagnosis of SCA is still challenging [22].

In the last few years, several technological advancements have been developed to improve the diagnostic performance of the standard imaging protocols. Even if novel technologies in CT, as photon-counting, appear to have a high contrast-to-noise ratio, randomized prospective trials are still required to assess their impact on diagnostic accuracy, especially in the differential diagnosis of rare pancreatic lesions. [23].Although AI tools have been integrated in radiological workstations for many anatomical districts, in abdominal imaging, they are limited to ultrasound examinations, prostate cancer, or 3D-reconstruction on CT images in pre-operative settings [24, 25].

### Limitations

This study has several limitations that must be taken into account. First, owing to the retrospective nature of this study, the relative proportions of the tumor histotypes included are unbalanced, as cystic pancreatic lesions less frequently underwent a CT examination; our results in terms of incidence of calcifications, therefore, could be unrepresentative of the true incidence of calcifications in pancreatic lesions. Second, we did not include PDACs and IPMNs, as calcifications within these tumors are exceedingly rare. Third, concerning image revision, further studies should be implemented, including the inter-operator agreement analysis for the classification of calcification.

## Conclusion

In conclusion, calcifications were observed in around 30% of pancreatic NENs, SCAs, MCNs, and SPNs. Intratumoral punctate calcifications appeared more common among NENs, while coarse intratumoral calcifications were more frequently encountered among SCAs. These patterns may help differentiate between tumor types, as coarse calcifications decrease the likelihood of NEN and increase the likelihood of SCA. However, given this study's retrospective and single-center nature, these findings should be validated in prospective multicenter cohorts.
